# Supplementary material for: Prunin Laurate Derived from Natural Substances Shows Antibacterial Activity against the Periodontal Pathogen Porphyromonas gingivalis
Source: Foods. 2024 Jun 18;13(12):1917. doi: 10.3390/foods13121917 (PMC11202431; doi:10.3390/foods13121917)
Supplement: Supplementary file 1 [file foods-13-01917-s001.zip › foods-2960490-supplementary.pdf]

Supplementary method

Time-kill assay

Suspension of *P. gingivalis* 33277 adjusted to a concentration of 1×10^8 CFU/mL in TSB broth was mixed with each food additive in a microtube. After incubation for 2 h, 4 h, and 8 h under anaerobic conditions, the bacteria were recovered by spinning down, washed with phosphate-buffered saline (PBS), resuspended, diluted to the limit, applied to TSB agar medium, and incubated under anaerobic conditions for 10 days. The bactericidal activity was determined by the number of bacterial colonies formed on the agar (Shinohara *et al.* 2022).

Shinohara M, Maetani M, Kitada C *et al.* Analysis of the Effects of Food Additives on *Porphyromonas gingivalis*. *Pathogens Basel Switz* 2022;**11**:65.

A

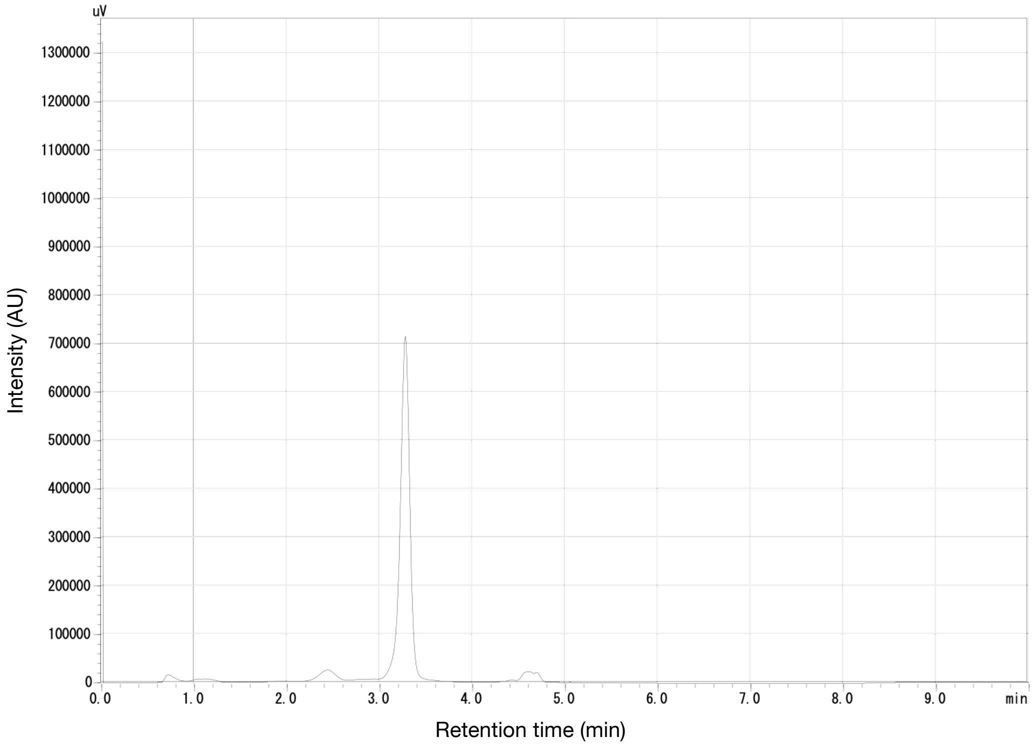

uV
1300000
1200000
1100000
1000000
900000
800000
700000
600000
500000
400000
300000
200000
100000
0
Intensity (AU)
0.0
1.0
2.0
3.0
4.0
5.0
6.0
7.0
8.0
9.0
min
Retention time (min)

B

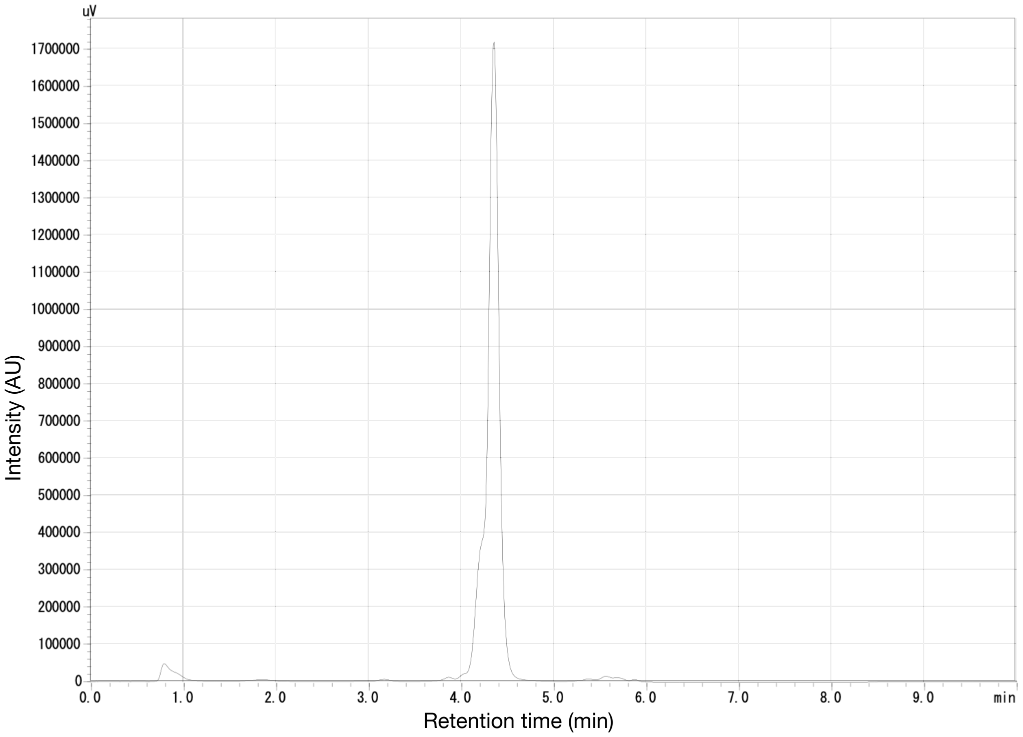
uV
1700000
1600000
1500000
1400000
1300000
1200000
1100000
1000000
900000
800000
700000
600000
500000
400000
300000
200000
100000
0
Intensity (AU)
0.0
1.0
2.0
3.0
4.0
5.0
6.0
7.0
8.0
9.0
min
Retention time (min)

C

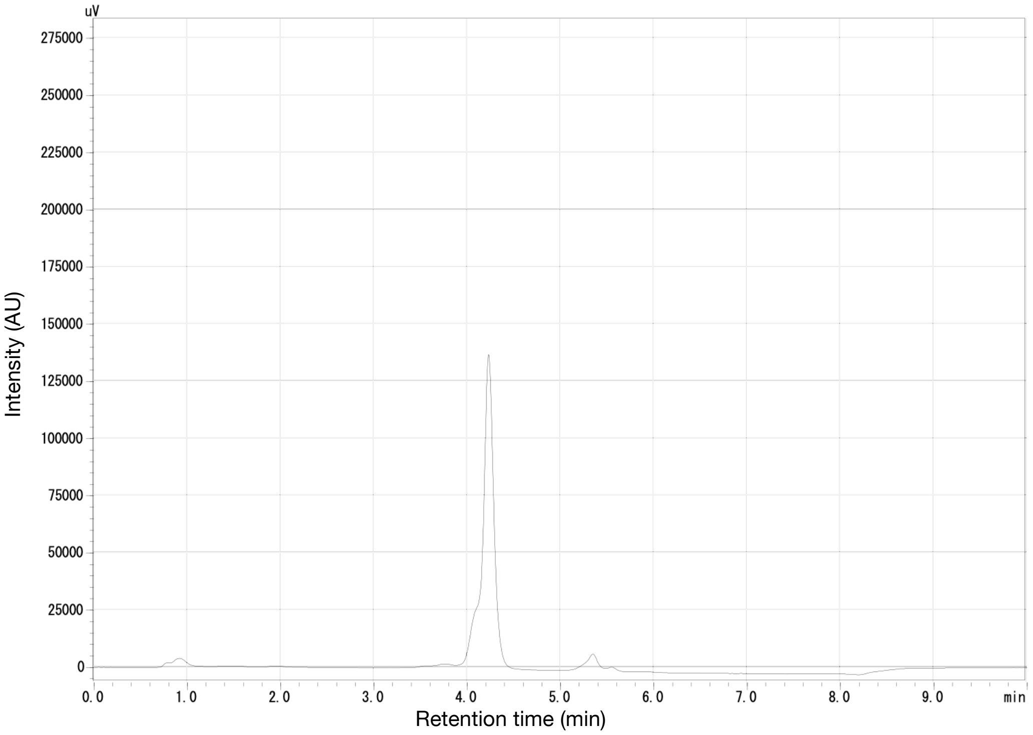
uV
275000
250000
225000
200000
175000
150000
125000
100000
75000
50000
25000
0
Intensity (AU)
0.0
1.0
2.0
3.0
4.0
5.0
6.0
7.0
8.0
9.0
min
Retention time (min)

D

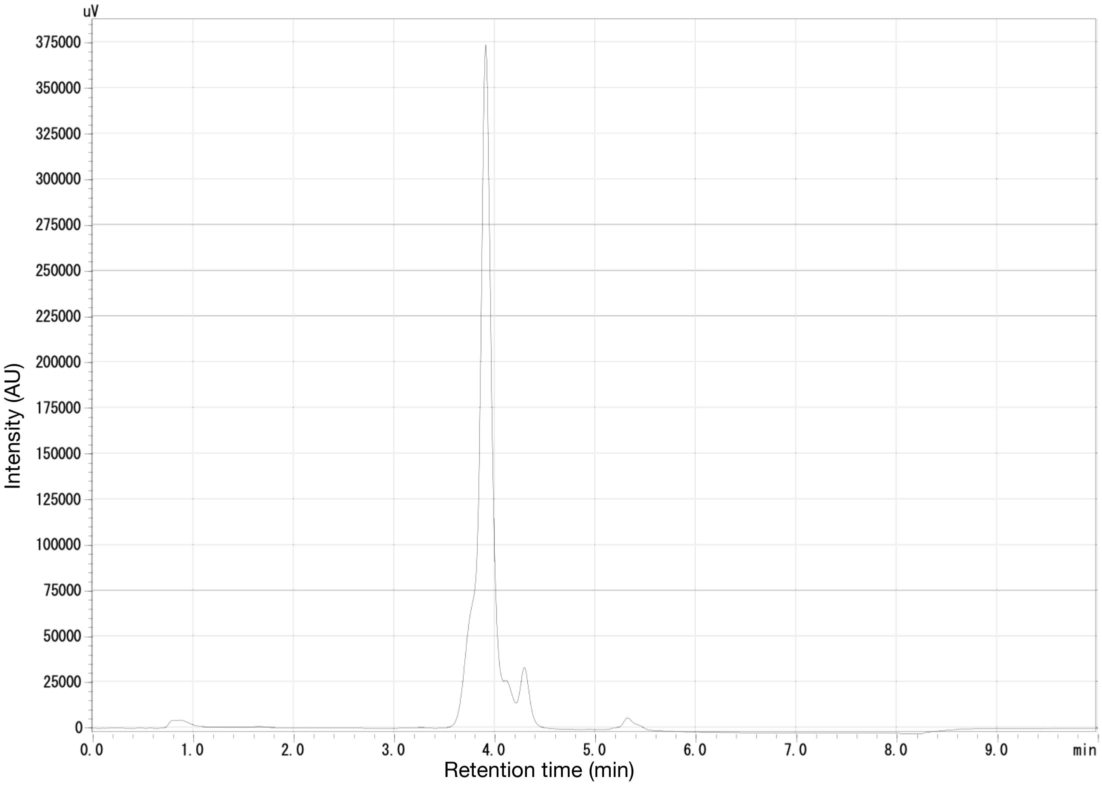
uV
375000
350000
325000
300000
275000
250000
225000
200000
175000
150000
125000
100000
75000
50000
25000
0
Intensity (AU)
0.0
1.0
2.0
3.0
4.0
5.0
6.0
7.0
8.0
9.0
min
Retention time (min)

E

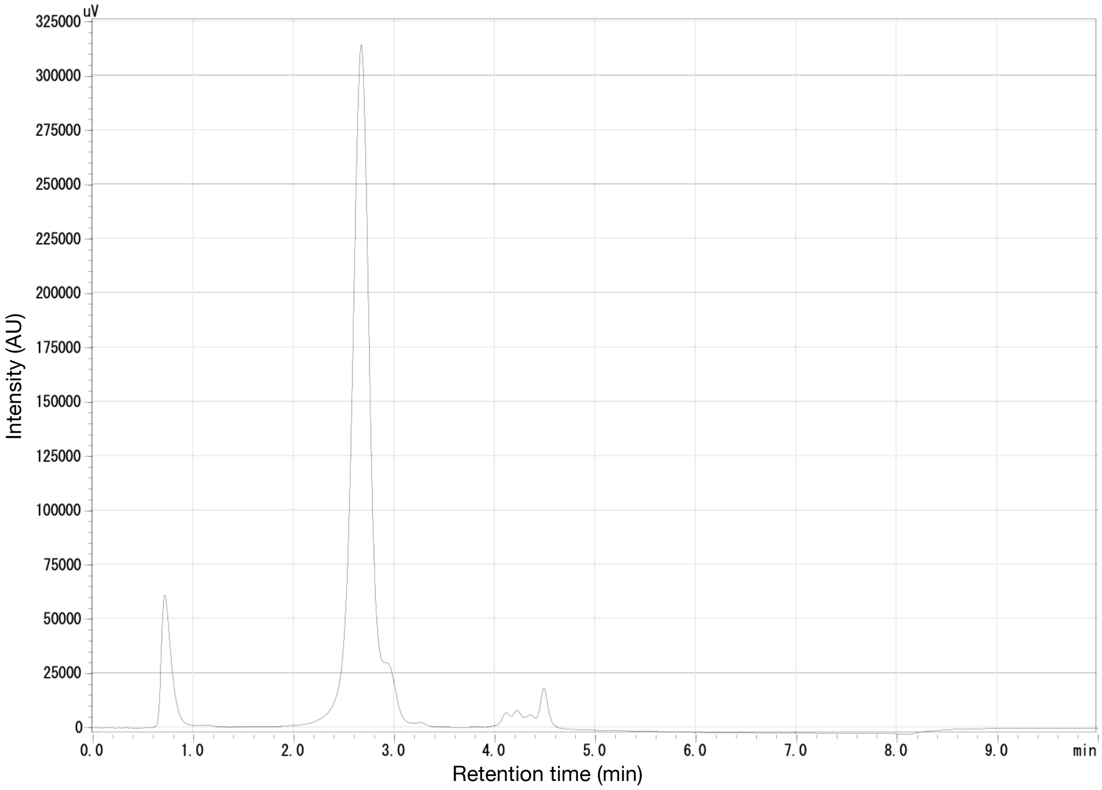
uV
325000
300000
275000
250000
225000
200000
175000
150000
125000
100000
75000
50000
25000
0
Intensity (AU)
0.0
1.0
2.0
3.0
4.0
5.0
6.0
7.0
8.0
9.0
min
Retention time (min)

F

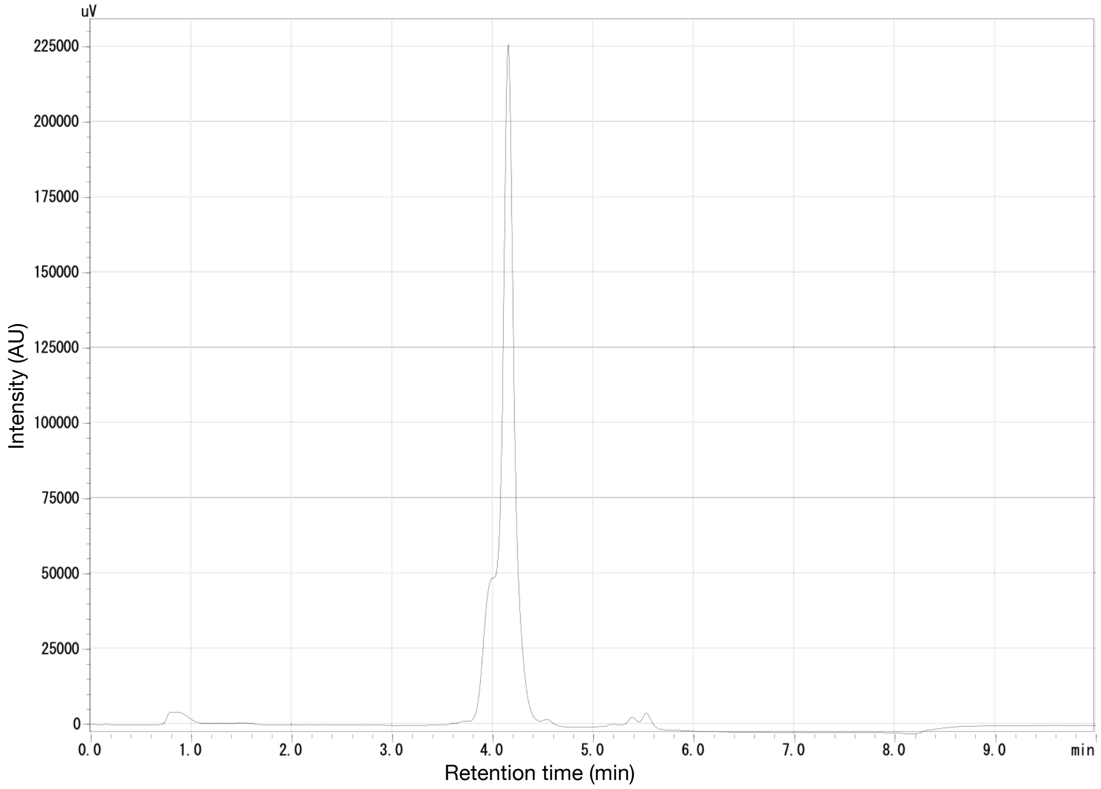
uV
225000
200000
175000
150000
125000
100000
75000
50000
25000
0
Intensity (AU)
0.0
1.0
2.0
3.0
4.0
5.0
6.0
7.0
8.0
9.0
min
Retention time (min)

G

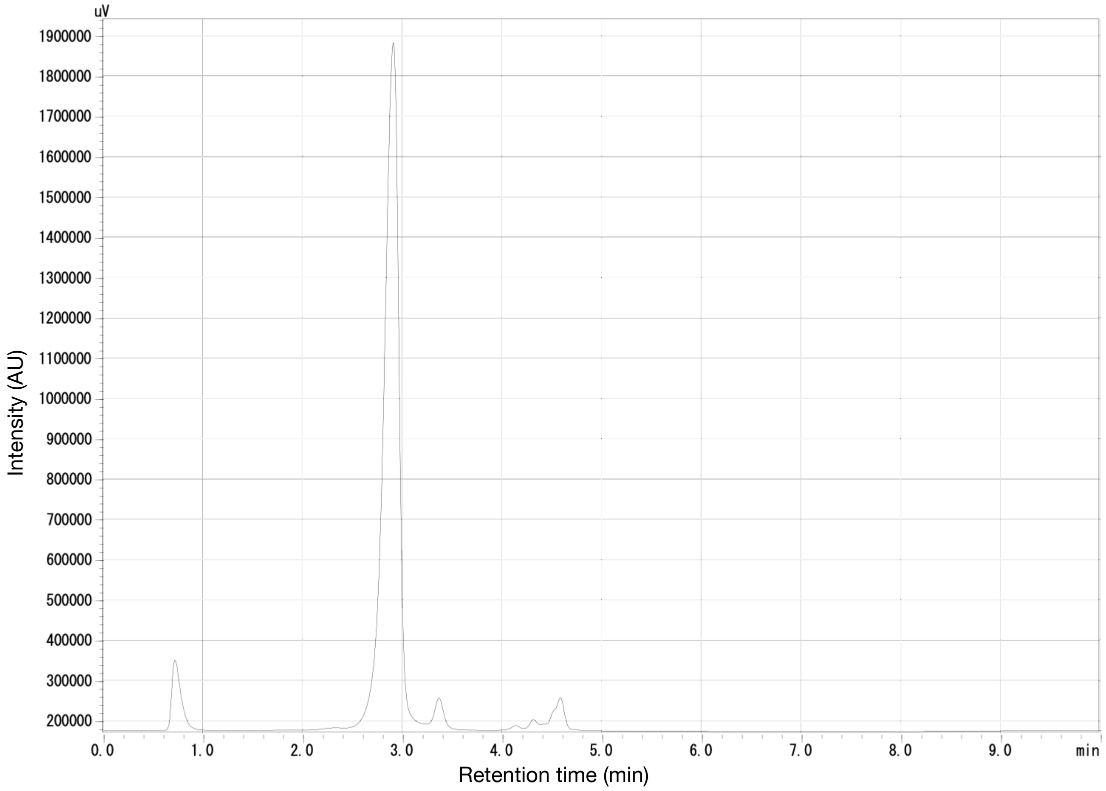
uV
1900000
1800000
1700000
1600000
1500000
1400000
1300000
1200000
1100000
1000000
900000
800000
700000
600000
500000
400000
300000
200000
Intensity (AU)
0.0
1.0
2.0
3.0
4.0
5.0
6.0
7.0
8.0
9.0
min
Retention time (min)

Figure S1. Purity of flavonoid lauroyl esters by HPLC chromatography

A: Pru-C12, Purity is 86%. B: Nar-C12, Purity is 96%. C: αG-Nar-C12, Purity is 91%. D: Rut-C12, Purity is 96%. E: αG-Rut-C12Purity is 86%. F: Hes-C12, Purity is 94%. G: αG-Hes-C12, Purity is 87%.

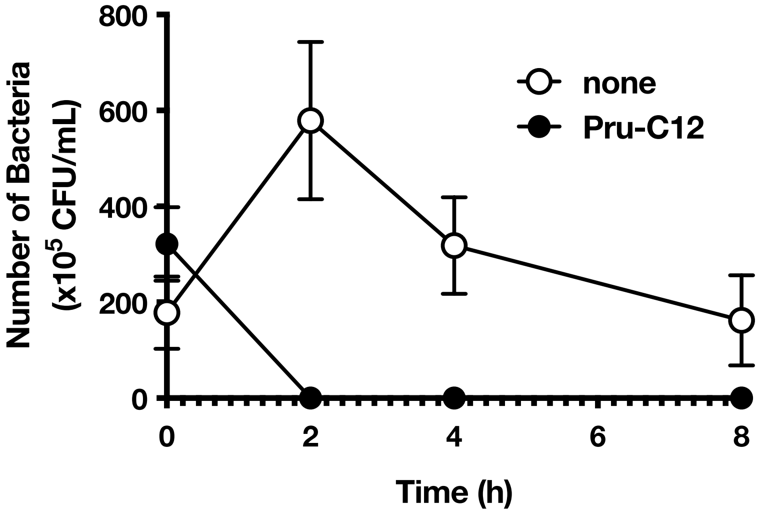

Figure S2. Time-kill assay of Pru-C12
